# Undergraduate medical education in Nigeria: current standard and the need for advancement

**DOI:** 10.11604/pamj.2021.40.40.30542

**Published:** 2021-09-16

**Authors:** Moyosoore Osoba, Shamsudeen Usman, Oluwafemi Oyadiran, Joseph Odeyemi, Michelle Abode, Olamide Usman, Olufemi Olulaja, Olusina Ajidahun, Don Eliseo Lucero-Prisno III

**Affiliations:** 1 African Center of Excellence for Genomics of Infectious Diseases (ACEGID), Redeemer's University, Ede, Osun State, Nigeria,; 2 St. Nicholas Hospital, Lagos, Nigeria,; 3 Medical Research Council Unit, The Gambia at London School of Hygiene and Tropical Medicine, Serrekunda, Gambia,; 4 Newday Specialist Hospital, Lagos, Nigeria,; 5 Hope Medical Center, Benin City, Nigeria,; 6 Crystal Specialist Hospital, Lagos, Nigeria,; 7 University of Ibadan, Ibadan, Nigeria,; 8 Department of Global Health and Development, London School of Hygiene and Tropical Medicine, London, United Kingdom

**Keywords:** Basic medical education, undergraduate medical curriculum, undergraduate medical training

## Abstract

The post-independence era in Nigeria ushered in an array of fundamental structuring and development in all sectors of the Nigerian economy including medical education and training. This era saw the establishment of medical schools across the country which mirrored the medical curriculum of British universities. This paper dives into the general structure of undergraduate medical education in Nigeria, its historical background and how it compares with neighboring and distant countries. Since the undergraduate medical education curriculum has not seen significant modifications since conception, this paper presents the challenges of the existent structure to include biased admission process, emphasis on irrelevant pre-medical courses, paucity of of technologically-advanced teaching and learning aids, increased workloads of lecturers amongst others. Importantly, solutions and recommendations are prescribed in this paper, which if considered, may improve undergraduate medical training in Nigeria, and ultimately improve the standard of healthcare service provision in the country.

## Essay

**General overview of medical education in Nigeria:** most Nigerian medical schools currently use a curriculum inherited from the British parent universities 60 years ago [1]. Undergraduate medical education in Nigeria has evolved over the years since it started in 1948 at the University College Hospital (UCH), Ibadan (a city in Southwestern Nigeria). Following this, majority of the recent medical schools embraced the curriculum of the earlier medical schools which was developed on the standards and curriculum identical to British universities, with minimal or no alteration [2]. From 1960 to 1972, medical schools were created at different parts of the country and medical education has since then expanded rapidly with the establishment of universities in almost every state in the country consisting of federal, state and private universities. The management of undergraduate medical education has continued to be under the oversight of the National Universities Commission (NUC) and the Medical and Dental Council of Nigeria (MDCN). Currently in Nigeria, there exists 42 medical schools of which 17 are federal, 18 are state, and 7 are privately owned [3]. Admission of students into the university is generally conducted by the Joint Admission and Matriculation Board (JAMB) and individual universities [4] via 2 pathways-university tertiary matriculation examination (UTME) for first year program applicants and Direct Entry (DE) for second year program applicants. Individual universities also conduct post-UTME exams to further screen candidates before they are eventually matriculated into universities.

Prior to writing the UTME and post-UTME examinations, it is required that prospective UTME candidates must have successfully completed the Senior Secondary Certificate Examination (SSCE) or General Certificate Examinations (GCE) as part of the prerequisite for admission [5]. The Bachelor of Medicine and Bachelor of Surgery (MBBS) degree program in Nigeria usually lasts a total of 6 years which is subdivided as follows: 12 months in preliminary basic sciences (which includes physics, chemistry and biology etc.), 18 months in the pre-clinical program in basic medical sciences where students take courses in human anatomy, medical biochemistry, human physiology, and the remaining 42 months taking courses in pathology and pharmacology before taking core clinical courses in medicine, surgery, obstetrics and gynecology, community medicine, and psychiatry [5]. On completion of the undergraduate program, medical graduates proceed to do a compulsory one-year internship and subsequently a mandatory one year of national youth service called the National Youth Service Corps (NYSC) in which doctors are mostly posted to remote areas for community services. This is constitutional and a pre-requisite for further continuance of medical education in federal or state institutions.

**Comparison of medical education in Nigeria with other countries:** medical education is quite similar in most African countries which follow a 6-year medical education system similar to the British-based curriculum of medical undergraduate training. The United States´ medical system incorporates a 4-year period called premed where courses are taken in the natural and applied Sciences like chemistry, physics, biology and depending on the institution, students may be advised to take additional advanced courses like molecular biology and genetics. In the United Kingdom, PreMed could last 5 years or 4 years whereas France´s system is divided into 3 cycles in which the first cycle lasts 2 years (during which students are taught basic natural and applied sciences; in addition, they learn foreign languages, epistemology, psychology, medical ethics and deontology) and the second cycle involves 4 years of intense pathology training, the last cycle which is for specialization is chosen by the student from a list of existing fields. Countries like the United States and the United Kingdom allow flexibility which gives room for combination of medical training with research or management programs called intercalated medical program. This allows graduates to have double degrees - (M.D/PhD), (M.D/MBA) etc, this however, is not seen in the Nigerian system of medical education. After completing undergraduate years, fresh graduates are awarded medical degrees in Nigeria. From this point, new doctors proceed to have a mandatory one year of internship (which is medical training continued with hands on) in accredited hospitals. In countries like South Africa and Ghana, internship is for two years. Doctors then proceed to the next step of specialization by applying for residency training programs.

### Challenges of undergraduate medical education

**Pre-medical school phase:** certain realities about the pre-medical school stage of undergraduate medical education in Nigeria posit as challenges and some of these include:

**Biased admission:** many aspiring and bright undergraduates looking forward to medical education get sidetracked by certain pre-existing catchment-based selection. This is usually either due to tribal preference of students hailing from the state in which the medical school is located or for reasons best known to people who have influence over the admission process [6]. The constant recurrence of this act of favoritism reduces the interest of some students while alienating others who have genuine passion to become doctors.

**Excessive admission:** the excessive admission of medical students over the years has had a negative impact on the medical education system in two main ways. Firstly, this overpopulation diminishes the quality of lecturing and learning. The available infrastructure and human resources can only cater for a certain number of students so as to meet minimum standards of training. The National Universities Commission (NUC) responsible for delivery of quality education in Nigeria has placed quotas on the admission of medical students into medical schools depending on variables such as staff strength, infrastructure and funds for renovation or expansion of facilities, amongst others. Yet, this quota and the requirements for a specific quota need to be reviewed and implemented as there is still evidence of overstretched resources in most universities. This may probably be due to poor adherence to the parameters used to access recommended quota in addition to the contributing factor of ineffective admission policies. This leads to an overwhelming situation both for the staff and the available resources for the pre-medical students which translates to poor learning environments and substandard education. Secondly, institutions which are well aware that they have exceeded their quota for admission into medicine cause students who do not meet a “created” cut-off at the first year (100 level) stage to be forced into unintended courses. This leads to a delay in the academic journey of those still willing to pursue medicine. It also causes psychological and emotional trauma to students and families after such students had been accepted at the UTME/POST-UTME stage to study medicine, only to be denied progression on their chosen career path.

**Poor departmental support:** the role of the faculty is to help students gain independence and confidence while creating a conducive environment for learning [7]. Students at the 100 level stage of pre-medical education are not under the supervision of the faculty of basic medical sciences and are left in the hands of other departments/faculties. As such, the support necessary to ensure a hitch-free learning process for medical students is lacking in pre-medical training in Nigeria.

**Cumbersome pre-medical foundation knowledge:** the task of teaching medical students at the pre-medical phase is known to be carried out by lecturers from faculties other than the Faculty of Basic Medical Sciences. Therefore, courses that are irrelevant to the introductory phase of medical learning have a tendency to be prioritized over more important aspects of medical education required to becoming a good doctor. This translates to medical students having to study irrelevant courses such as botany during pre-medical education.

**Medical school phase:** the challenges for education of the best possible physicians are great but the benefits to medicine and society are enormous [8]. While this is the case, the pre-clinical and clinical phases of medical training in Nigeria are also laced with challenges.

**Relying on the other faculties to teach basic medical sciences:** in the same vein as pre-medical education which is taught by other faculties, basic medical science courses are also often taught by non-doctors. The foundation of medical knowledge is the basic medical sciences, hence to maximize retention and subsequent application of this knowledge, it has to be taught in a clinically-oriented manner. Medical education is a specialty on its own and most people who teach it are not formally trained to [9]. This leaves a defect in the quality of performance of medical students in the clinical class and subsequently during residency because the basic medical sciences were not taught in a clinically-oriented manner.

**Dated medical curriculum:** the medical curriculum is not updated and as such, many new scientific developments and technological advancements may be alien to medical students who eventually do their residency program in developed countries. Of great concern is the fact that graduates from medical colleges in Nigeria may lack the necessary skill and aptitudes required for success in the changing practice environment of the 21^st^ century [10]. To adapt in foreign climes, most Nigerian-trained doctors have to unlearn old practices and learn conventional techniques to function effectively.

**Inadequate learning infrastructure:** the Nigerian system of medical education scarcely employs the use of nascent teaching aids such as mannequins, role play, simulations etc. This hinders practical learning and can impair the understanding of students since they need to rely on their imagination to understand some of the concepts taught.

**Overburdening the clinical teacher:** the clinical teacher is the substructure on which the medical system is built. However, the quality of the clinical teacher is diminished due to excess burden as the teacher is expected to teach, deliver medical care to patients, carry out research and other administrative responsibilities under poor conditions. In addition to these, the clinical teacher is inadequately remunerated despite often undefined working hours. These result in a psychologically and physically drained teacher who is not motivated to teach, or at best; teach at sub-optimal potential.

**Differing management authorities for medical schools and teaching hospitals:** some medical schools and their accompanying teaching hospitals are managed and funded by different authorities creating a conflict of interest in funding of clinical training and medical research [11]. For example, compensation for hospital consumables used for research, teaching and examinations may become a topic of dispute between a medical college and its hospital authorities. This poses challenges in teaching, research and utilization of facilities. Consequently, learning will be deterred or alternatively the medical student will be burdened with the responsibility to provide himself the materials needed. These factors will contribute to making the medical student despondent about acquiring the required knowledge to become a well-trained doctor.

**Professional bullying:** it is no news that hierarchy exists in the medical profession; bullying has gradually become a normal way of training medical students. Bullying of medical students was systematically studied and the association of American medical colleges graduation questionnaires (AAMC GQs) from 2012 and 2013 reported students´ mistreatment rates of 47.1 percent and 42.1 percent, respectively [12]. This increases the psychological stress on the already overburdened student and consequently deters learning and ultimately patient care.

**Assessment of medical students:** medical education is broad and thus it is difficult to adequately assess clinical knowledge. However, Objective Structured Clinical Examination (OSCE) was introduced by Harden in 1975 as an alternative to the existing methods of assessing clinical performance; [13] it uses simulated clinical conditions to assess the skills of medical students. Although this method is objective and evaluates the cognitive and psychomotor domains of the medical students, it has failed to assess the ethical reasoning as well as communication skills of medical candidates [14] which are vital qualities of a medical doctor. This consequently contribute to producing doctors with poor interpersonal skills, poor ethical considerations and poor empathetic approach to patient management.

**Grading system:** although there is no perfect grading system, the current grading system which utilizes negative marking (points are deducted for every wrong answer from points earned for right answers) makes students unnecessarily anxious [15] and ultimately impacts negatively on the transcript of many medical graduates putting them at a disadvantage in the global community when competing for scholarships and other grants.

**Inadequate research exposure:** Nigeria utilizes research findings from developed countries and while some of these research findings have promoted health care advancements, others have failed to do so as the findings are not applicable locally [16]. In most medical schools in Nigeria, students are exposed and supervised to carry out research projects for the first time during their final year of study as part of compulsory graduation requirements. Hence, many students don´t glean the required knowledge and are unable to hone their research skills in such short period as most of them perceive these projects as prerequisites for graduation instead of a fundamental part of medical education. This invariably breeds clinicians with subpar research skills and limits medical advancement in the country.

**Poor attention to mental health:** medical training is rigorous and asides the high level of stress in the medical school, students have to grapple with insecurity, social problems and poverty among others with little or no support from the university. A systematic review revealed that perceived stress, depression and use of psychoactive substances among medical students in Nigeria were as high as 60.5%, 33.5% and 44.2% respectively. Prevalence of psychiatric disorders is high among Nigerian medical students and many of these students transform into doctors who pay little to no attention to the mental health of their junior colleagues and subordinates [17].

**Absence of intercalated degrees:** high-flying medical students are not given the option of obtaining intercalated medical degrees like is found in developed climes. Hence, students with stellar academic records and achievements are subjected to the same generic training as their peers and this ultimately puts some at a disadvantage when competing for global opportunities with their counterparts from other climes that possess these intercalated degrees.

**Possible solutions to the challenges of undergraduate medical education in Nigeria:** as a program designed to prepare doctors to deliver healthcare to the community and one which is a determinant of the progression of a country´s health care system, [18] identifying and providing solutions to the defects in undergraduate medical training is very important. The following are possible solutions to the challenges facing undergraduate medical education in Nigeria.

**Revise admission criteria:** as one of the most ethnically diverse countries in the world with over 250 different ethnicities, [19,20] it is understandable that achieving equity in the educational and health systems just like every other sector is constantly a topic of discussion. While the inclusion of catchment areas and ethnical bias to the admission process may seem like it gives some people a better chance [21], it takes us farther from the equity we desire. A much more efficient and fair intervention will be addressing the structural problems and systematic disparities that prevent individuals living in certain regions from being able to meet up with the academic requirements for medical training in the university. These issues include ignorance, distrust of western education, poor quality of primary and secondary education and poverty [22]. Schools should be required to use similar admitting standards for both indigenes and non-indigenes while scholarship offers can be introduced to encourage the under-represented ethnic groups. This will ensure that the most deserving and passionate students get admitted to study medicine. Furthermore, this will increase the frequency of students studying out of their home states with the possible impact of promoting a more even distribution of medical doctors nationwide.

**Revise medical curriculum:** the currently congested medical curriculum needs to be modified and streamlined with an increased clinical focus. In the pre-med classes, courses like practical biology, medical physics (a branch of applied physics) and medical biochemistry need to be given a larger share of the modules while theoretical physics and botany need to be downplayed. The current syllabus in pre-med biology is very similar to what was taught in secondary school, with a large part focusing on plants and animals. This course needs to be modified [23] into an introductory class to human biology to give prospective medical students an idea or foundational knowledge to prepare them more adequately for medical school. Furthermore, the basic medical sciences are not taught in a clinically-oriented manner since majority of the lecturers in this faculty are not medical doctors and teach from their perspectives as anatomists, biochemists and so on [8]. The solution to this will be either hiring medical doctors with graduate training in these subjects to lecture medical students or to require some additional training in the clinical aspects of these subjects from non-doctors [24]. This will ensure a more seamless transition from the basic medical sciences into the clinical classes and will also encourage medical students to develop a clinical perspective early in their studies.

Also, while it is necessary for the medical curriculum of any country to be streamlined to the predominant medical conditions and available medical technology, Nigeria is lagging in terms of technological developments that some of the treatment plans in our medical curriculums are archaic [8]. The medical curriculum needs to be updated to include the latest scientific breakthroughs and technological advancements. Lastly, in the Nigerian system, the doctor typically finds himself as the administrative head of hospitals and health centers and so courses that teach leadership and management skills should be added to the curriculum [8]. Furthermore, research should be introduced much earlier in undergraduate medical education and students should be mentored in this regard and encouraged to publish projects. This will invariably lead to good research practices among doctors, medical advancements and adoption of evidence-based clinical practice among clinicians. Also, intercalated medical programs should be introduced as an option for high-flying medical students, as this will help maximize their potential and put them at par with their counterparts in other climes.

**Infrastructural development:** substantial investments need to be made towards the procurement of medical infrastructure and learning tools like computers and mannequins which are lacking or insufficient in most medical schools in Nigeria [24]. This will promote practical teaching and learning and markedly improve the understanding of medical students. Political will is required in this regard as around 90% of medical schools and teaching hospitals in Nigeria are government owned [25]. The government needs to spend to improve the state of the nation´s medical schools and tertiary health institutions [25].

**School structure and values:** teachers need to encourage active learning and critical thinking by utilizing class discussions, study groups and assignments that encourage research and thoughtful reflection. An example of this is problem-based learning, where students learn by applying critical thinking to solve open-ended problems [26]. These should be scored and included in the grading process so that passing or failing is not dependent on an absolute cut off mark set for one test as this stifles creativity and encourages mechanical studying-cramming [18,23]. Secondly, medical schools should ensure that a mini-department or at least a member of staff is assigned to pre-med students to provide counsel and direction especially with administrative issues as they are often isolated from the rest of the medical school [27]. Also, some of the current methods for assessing medical students that focus on speed of recollection rather than proper understanding, communication and ethical skills need to be down-played. An example is the objectively structured clinical examination (OSCE) which has reported serious flaws, [28] stations during these clinical assessments should be longer and should assess students on effective communication, empathy and ethics asides clinical knowledge.

**Attention to mental health:** with the prevalence of psychiatric illnesses among medical students, easily accessible mental health services should be core a part of student welfare scheme. Medical schools should have easily accessible psychiatrists and counsellors dedicated to the students. Furthermore, schools should also prioritize holidays as these will promote academic de-escalation and reinvigoration of medical students.

**Academic staff welfare and inter-organizational synergy:** the government needs to show more interest and better fund teaching hospitals [23]. It is important that medical schools and teaching hospitals are adequately staffed and that staff salaries are satisfactory and promptly paid. This will improve the ability and motivation of professors to teach medical students [8]. The federal and state governments need to ensure that hospital funding is constant and not affected by the bureaucracies of budgeting and disbursement of funds [25]. Also, bullying in medical school needs to be clamped down on. Human resource departments need to be held accountable, they need to take action when students make complaints of bullying and harassment by the teachers and not just ignore [29]. Furthermore, creating a nexus between the management of medical schools and affiliated teaching hospitals is key to ensuring high standard undergraduate medical training.

## Conclusion

Despite the many challenges associated with undergraduate medical training in Nigeria, the prevalent system is not without its merits. However, an improvement in the selection criteria, curriculum, structure of training, infrastructure, teaching aids are prerequisites needed to be instituted if medical training in Nigeria will be at par with contemporary standards. While this will require political will on the part of federal and state governments, medical elders also need to realize and vanguard the need for transitioning from the existent inherited system of training to that consistent with the rapidly evolving field of medicine. This will in turn not only abate the hurdles which have plagued undergraduate medical education in Nigeria, but also lead an overall upgrade in the quality of healthcare service delivery in the country.

## References

[1] Federal Ministry of Health of Nigeria (2012). Nigerian undergraduate medical and dental curriculum template.

[2] Ibrahim M (2007). Medical education in Nigeria. Med Teach.

[3] Medical and Dental Council of Nigeria (2006). The red book medical and dental council of Nigeria guidelines on minimum standards of medical and dental education in Nigeria.

[4] Adeniyi K, Sambo DU, Anjorin FI, Aisien AO, Leonard R (1998). An overview of medical education in Nigeria. J Pa Acad Sci.

[5] Malu AO (2010). Universities and medical education in Nigeria. Niger Med J.

[6] Nto NJ, Obikili EN, Anyanwu GE, Agu AU, Esom EA, Ezugworie JO (2019). Effect of age, premedical academic performance, and entry bias on students' performance in final preclinical examination at the University of Nigeria Medical School. J Exp Clin Anat.

[7] Olujide O (2005). Redefining undergraduate medical education in the College of Medicine University of Ibadan: the views of a medical student. Archives of Ibadan Medicine.

[8] Ezeanolue B (2011). Current challenges of medical education in Nigeria. Int J Med Health Dev.

[9] Mckimm J (2009). Professional development for medical educators and clinical teachers: challenges and opportunities. South East Asian J Med Ed.

[10] Silver H, Glicken A (1990). Medical student abuse: incidence, severity, and significance. JAMA.

[11] Olasoji H (2014). Rethinking the approach to curriculum review in medical and dental education in Nigeria. J Ed Pract.

[12] Association of American Medical Colleges (2017). Medical school graduation questionnaire: all schools summary report 2017.

[13] Harden RM, Stevenson M, Downie WW, Wilson GM (1975). Assessment of clinical competence using objective structured examination. Br Med J.

[14] Sholadoye T, Tolani MA, Aminu MB, Maitama Hussaini (2019). Clinical examination among medical students: assessment and comparison of the strengths and weaknesses of objective structured clinical examination and conventional examination. Niger J Surg.

[15] Ndu I, Ekwochi U, Di Osuorah C, Asinobi IN, Nwaneri MO, Uwaezuoke SN (2016). Negative marking and the student physician: a descriptive study of Nigerian medical schools. J Med Educ Curric Dev.

[16] Awofeso O, Roberts AA, Okonkwor CO, Nwachukwu CE, Onyeodi I, Lawal IM (2020). Factors affecting undergraduates´ medical research in Lagos. Niger Med J.

[17] Esan O, Esan A, Folasire A, Oluwajulugbe P (2019). Mental health and wellbeing of medical students in Nigeria: a systematic review. Int Rev Psychiatry.

[18] Scheele F (2012). The art of medical education. Facts Views Vis Obgyn.

[19] Fearon J (2003). Ethnic and cultural diversity by country. Journal of Economic Growth.

[20] Central Intelligence Agency (CIA) Explore all countries-Nigeria.

[21] Omeje J, Egwa E, Adikwu V (2016). Impact of quota system and catchment area policy on the university admissions in North Central Nigeria. SAGE.

[22] United Nations Children's Fund (UNICEF): Nigeria (2013). The challenge: one in every five of the world’s out-of-school children is in Nigeria.

[23] Akubuilo F, Okorie E (2013). Sustainability of tertiary education through quality assurance and development in Nigeria. J Educ Pract.

[24] Swanwick T (2008). See one, do one, then what? Faculty development in postgraduate medical education. Postgrad Med J.

[25] Alkali N, Bello M (2020). Tertiary hospital standards in Nigeria: a review of current status. Annals of African Medical Research.

[26] Preeti B, Ashish A, Shriram G (2013). Problem based learning (PBL); an effective approach to improve learning outcomes in medical teaching. J Clin Diagn Res.

[27] Oladipo A, Adeosun O, Oni A (2009). Quality assurance and sustainable university education in Nigeria. Hiroshima University.

[28] Asani M (2012). Assessment methods in undergraduate medical schools. Niger J Basic Clin Sci.

[29] Owoaje E, Uchendu O, Ige O (2012). Experiences of mistreatment among medical students in a university in south west Nigeria. Niger J Clin Pract.

